# New anti-neoplastic drug test using in vitro model: what to be concern?

**DOI:** 10.1186/2008-2231-20-65

**Published:** 2012-10-24

**Authors:** Viroj Wiwanitkit

**Affiliations:** 1 Hainan Medical University, Hainan, China; 2 Joseph Ayobabalola University, Abuja, Nigeria; 3 Faculty of Medicine, University of Nis, Nis, Serbia; 4 Wiwanitkit House, Bangkhaae, Bangkok, Thailand

**Keywords:** Anti-neoplastic drug, Model, Test

## 

An important medical disorder that is common but not successfully managed in the present day is cancer. In fact, cancer has been known in medicine for many centuries. Continuous attempt to find anti-neoplastic drug leads to several ant-cancer drugs at present. However, as noted, the actual effective drug is still not available. Hence, cancer is still a deadly disease. Finding for new anti-neoplastic drug is the focus in cancer research at present. In the process, after developing of new drug alternatives, the next step is to study on its efficacy. Basically, the evaluation on pharmacological and toxicological effects has to be done for confirming the effectiveness and safety of the new drug. To serve this purpose, the in vitro model study is generally gone as the pioneer step. It is also the basic requirement for further drug registration [1]. Focusing on the vitro model study, there are some facts to be addressed. First, the model is not an actual thing. It is only the attempt to simulate the real phenomenon. Second, the study is generally in vitro. This is for the safety reason. In vitro study means the experiment outsides the living things. Hence, an in vitro study is totally not the real case in human beings. In general, the better study can be done based on in vivo study only if the safety is completely confirmed. It should be further noted that in vivo study can also be either in animal model or real human subjects. The best interpretation has to be based on finding for human study. Nevertheless, the human study has the highest risk comparing to the other techniques. Focusing on the new anti-neoplastic drug, it is perceived as a dangerous chemical agent. Hence, it is rarely studied in human subjects. Also, it is very hard to include cancerous patients to enroll in such study and it should be unethical to perform such experiment in normal subjects. Furthermore, any studies have to follow the good research guideline. The use of randomized control trial is usually preferable for final acceptance on registration process [2]. Finally, the present in vitro model study also poses the pitfall. Most in vitro model for the novel anti-neoplastic drug test is based on the cell culture, which is usually transformed neoplastic cell type [3]. Those transformed cell is not normal physiological cells, hence, the normal physiological response to the studied chemical agent cannot be expected. Those cancerous cells might not respond well or tolerate to side effect [3].

Considering the present in vitro cell culture study for new anti-neoplastic agent, several facts should be discussed. First, as already noted, the observation on the drug study is derived from abnormal transformed cells which usually passed several biomedical modification and sub-culture process. This cannot directly and totally imply the normal cells and non transformed, non sub-cultured cancerous cells. To refer for effectiveness and safety for further human study or usage must be careful.

Second, in vitro model cannot include any biotransformation that might occur in vivo. Drug distribution, drug elimination and interference from other biochemical substances in human body are all not studied in the vitro test. Third, it should be noted that several new anti-neoplastic alternative are developed with advanced biomedical engineering technology [4], such as recombinant protein technology and nanopharmacology. Since the medical society still has few information on the side effect of those new substances (new recombinants, new nanomaterials, etc.). The complete study on the toxicological effect is required. For the new nano-anti-neoplastic drug, the drug in vitro might totally not reflect the actual action in human body. Since the nanomaterial is highly sensitive, it might aggregate to form larger molecule in vivo or it might interact with several substances, especially for proteins, in human body. For sure, if such scenario occur, the change in pharmacological and toxicological properties is possible. The development of new drug test system for nanomaterial – based anti-neoplastic drug is required and the test must be performed within certified nanotechnology laboratory. Fourth, it must be repeatedly noted that anti-neoplastic agent is chemical hazardous. The safety concern should be for not only the patients but also on the medical personnel who develop and perform drug test. The safety of the practitioners seems to be the forgotten issue. Finally, the new alternative mean to test the new drug should be mentioned. With advanced computational technology, the use of bioinformatics tool to help simulate and study on the drug pharmacology and toxicology is possible.

## Competing interests

The author declares that he has no competing interests.

## Authors contributions

VW, concept, draft, final check (100%).
